# Children’s Expectations and Understanding of Kinship as a Social Category

**DOI:** 10.3389/fpsyg.2016.00440

**Published:** 2016-03-29

**Authors:** Annie C. Spokes, Elizabeth S. Spelke

**Affiliations:** Laboratory for Developmental Studies, Department of Psychology, Harvard University, CambridgeMA, USA

**Keywords:** social cognition, kin preference, development, social categories, resource sharing

## Abstract

In order to navigate the social world, children need to understand and make predictions about how people will interact with one another. Throughout most of human history, social groups have been prominently marked by kinship relations, but few experiments have examined children’s knowledge of and reasoning about kinship relations. In the current studies, we investigated how 3- to 5-year-old children understand kinship relations, compared to non-kin relations between friends, with questions such as, “Who has the same grandmother?” We also tested how children expect people to interact based on their relations to one another, with questions such as “Who do you think Cara would like to share her treat with?” Both in a storybook context and in a richer context presenting more compelling cues to kinship using face morphology, 3- and 4-year-old children failed to show either robust explicit conceptual distinctions between kin and friends, or expectations of behavior favoring kin over friends, even when asked about their own social partners. By 5 years, children’s understanding of these relations improved, and they showed some expectation that others will preferentially aid siblings over friends. Together, these findings suggest that explicit understanding of kinship develops slowly over the preschool years.

## Introduction

Humans categorize people as members of multiple groups, based on diverse commonalities including family, race, religion, ethnicity, economic class, and gender. We form social categories even in situations in which groups are arbitrary or randomly assigned (Tajfel et al., 1971). Recent research reveals that age, gender, race, ethnicity, and language are salient social categories for infants and young children, who show preferences for members of their own group (e.g., Kinzler et al., 2010). Nevertheless, one important social distinction has received little attention in current investigations of children’s social cognitive development, despite its social importance for children worldwide: the distinction between kin and non-kin.

Investigators in anthropology, sociology, and human biology have explored the rich dynamics of familial relations. According to long-accepted principles of evolutionary theory, individuals achieve indirect benefits to their inclusive fitness when their kin survive and reproduce (Hamilton, 1964; cf. Nowak et al., 2010). Thus, humans could be predisposed to track and help kin members. Consistent with this theory, human adults encoded kinship to the same extent as sex and age in a memory confusion paradigm (Lieberman et al., 2008), and they show evidence of a kin detection mechanism that influences opinions and behaviors related to sibling altruism and incest disgust (Lieberman et al., 2007).

Little research has explored children’s knowledge and reasoning about kinship relations, however, and most experiments that have done so suggest that sensitivity to kinship develops slowly. At 5 years, children are apt to apply kinship terms to people on the basis of their typical perceptual features rather than their kinship relations (Landau, 1982). For example, 5-year-old children, asked to determine which person is a “grandmother,” typically chose a person who looked old but was pictured without children and grandchildren over a person who looked younger but was pictured with children and children’s children (Landau, 1982). Furthermore, children first demonstrate a clear understanding of biological knowledge of life and death between the ages of 5 and 7 years (Carey, 1985; Inagaki and Hatano, 2002), so the underlying biological nature of something like blood relations may not develop until late in childhood. Nevertheless, children may have earlier intuitions about the social nature of kin relations.

In addition to understanding the meaning of different social relations, children need to understand and make predictions about how people will interact with one another, in order to navigate the social world. One domain that is central to human social relations and cooperation is resource sharing. Even before age two, infants are biased toward equal distributions of resources (Schmidt and Somerville, 2011; Sloane et al., 2012; Somerville et al., 2012). Children tend to share resources with others equally when they are able to (e.g., Olson and Spelke, 2008), taking into account the value of a resource when calculating equality (Shaw and Olson, 2013). By age six, children tend to dislike those who do not share equally (Shaw et al., 2012). Nevertheless, children override a preference for equality and accept unequal resource distributions when there is evidence that the recipient is more deserving due to prior behavior or social group status (e.g., Sloane et al., 2012).

Moreover, both adults and young children expect others to share resources according to principles of direct and indirect reciprocity (Wedekind, 2000; Wedekind and Braithwaite, 2002; Greiner and Levati, 2005; Gurven, 2006). Adults have demonstrated a bias to work harder in order to benefit others more closely related to them (Madsen et al., 2007), although young children have not shown a clear preference to benefit kin (Olson and Spelke, 2008). In these studies, 3.5-year-old children were introduced to dolls that were siblings, friends, or strangers with a protagonist doll and helped the protagonist divide up nine resources across trials. Children tended to give more to those who had shared with the protagonist or with others previously, providing further evidence of their sensitivity to direct and indirect reciprocity. Children also gave more to the protagonist’s siblings or friends than to strangers, but they gave roughly equally to siblings and friends. However, resources were plentiful enough to be distributed to everyone, and they were relatively low in value (Olson and Spelke, 2008).

Here we investigate further how children understand the relation between siblings as compared to friends or strangers, and how they expect people to interact based on their relations to one another. Will children distinguish among close relations when they must divide up resources of lower availability and therefore higher value? Furthermore, will children demonstrate different sharing behavior when distributing resources among their own close relations rather than in hypothetical, third-party scenarios, or when presented with realistic photographs of faces showing a strong family resemblance rather than with dolls? Finally, will children’s understanding track with their expectations for social interactions?

We hypothesize that children may be able to distinguish kin and non-kin from a young age. To test this, we present children with social scenarios using verbal presentation in storybooks and ask explicit questions about their understanding and sharing behavior. We examine whether children show sensitivity to kinship distinctions or whether this sensitivity may not emerge until later in development in these scenarios.

In Experiment 1, we tested 3- and 4-year-old children’s conceptual understanding and resource sharing choices for fictional characters in a storybook and examined whether their predictions of sharing toward kin, friends, and strangers were influenced by the value of the resource. In Experiment 2, we tested whether children distinguish kinship from friendship when asked about their own siblings and friends, and we expanded the age of children tested to include 5-year-old children. In Experiment 3, we further investigated 3-, 4-, and 5-year-old children’s expectations for social interactions, using physical similarity to enhance kinship cues and probing children’s social inferences across a more diverse set of social contexts. Taken together, these studies shed light both on 3- to 5-year-old children’s conceptual understanding of kinship and on the ways in which their prosocial decisions are, and are not, affected by kinship.

## Experiment 1

The first study investigated children’s expectations for sharing of resources in third-party social interactions among kin, friends, and strangers. The resources varied in value, based on their plenitude or scarcity. This study also tested children’s conceptual understanding of siblings, friends, and strangers. An experimenter read children an interactive storybook in which a protagonist character traveled to different locations and interacted with her sibling, her friend, and a child whom she had never met before. Children were asked factual questions about the different characters to probe their understanding of these social relationships. Then, they were asked to predict with whom the protagonist would share a resource.

### Materials and Methods

#### Participants

Ninety-six children from the Cambridge and Boston area participated in this study, with 48 children at each of two ages: 3.5 years (26 female, 36.23–47.43 months, mean age = 41.46 months) and 4.5 years (24 female, 48.27–60 months, mean age = 53.39 months). Children received a gift after their study, and parents were reimbursed for their travel. This study was carried out in accordance with the recommendations of the Committee on the Use of Human Subjects in Research at Harvard University with written informed consent from a parent or legal guardian of all subjects and verbal agreement from participants.

#### Materials

We presented participants with fictional characters in a storybook using all hand-drawn, cartoon-like pictures colored with marker. Each story focused on one protagonist who interacted with her sister, friend, and a stranger in different scenarios. The storybook consisted of one warm-up scenario followed by three scenarios each composed of an introductory questions phase and a sharing phase. Thus, children were introduced to four scenarios: one warm-up with animal characters and three test scenarios with human characters and social interactions. In the introductory phase, children were shown the protagonist in a new location, with two other characters. The other characters were described and named, and their relation to the protagonist was indicated: a sister (henceforth, sibling), friend, or a girl she had never met before (henceforth, stranger). Children’s understanding of the relations then was probed through two questions focused on the two characters’ relationship (described below).

In the sharing phase, participants were introduced to a scenario in which the protagonist now had a valuable resource (e.g., a trip to the beach) to share with one of the two other characters. In all contexts, there was only one resource, so the protagonist could only share with one of the two characters. Children were given drawings symbolizing the resources to be distributed in the storybook: a green pet toy, a cupcake, a seashell, and a banana. The same storybook was used across resource conditions, but the drawings were described as representing differently valued objects across conditions.

#### Procedure

Children were first told they were going to hear a story about a protagonist, Cara, and her adventures. Then children began the warm-up phase of the study, in which the protagonist was presented with two animals: a dog and a cat. The experimenter asked children two questions about the animals (“Which one likes to play fetch?” and “Which one purrs when you pet it?”). Children were always given positive reinforcement for their answer, whether correct or incorrect. After these questions, the next page in the storybook showed the protagonist with a resource. The warm-up involved a pet toy that was described as being liked by both cats and dogs. The experimenter then gave the child the drawing of the toy and encouraged the child to help the protagonist decide which animal to give it to. Children were encouraged to place the item in front of the animal they chose. If children wanted to give the resource to both animals, they were told to choose one since they only had one toy to give.

After the warm-up phase, the story advanced to the first test block involving people interacting with the protagonist. Each block began with the introductory phase, which first showed the protagonist in a new location: the park, the beach, or the zoo. Children were encouraged to discuss activities for the protagonist to do at the new location in order to keep them engaged in the storybook. The next page in the storybook for each block showed the protagonist with two other characters described as her friend and sibling, friend and stranger, or sibling and stranger. The warm-up phase always came first, but the order of the three social scenarios and pairs was counterbalanced across participants.

After children were introduced to the pair of characters, they were asked two questions about their specific relations to the protagonist. For friend and sibling, the questions were: “Which girl has the same grandparents as Cara?” and “Which girl could Cara meet for the first time at school?” The questions for friend and stranger were: “Which girl has Cara played with many times before?” and “Which girl does Cara not know much about?” For sibling and stranger, children were asked, “Which girl has the same last name as Cara?” and “Which girl has Cara never seen before?”

The next page in the storybook for each block showed the protagonist with a newly acquired resource, as in the warm-up phase. The displayed picture was the same across conditions, but the resource was described differently based on whether the condition is a high- or low-value resource condition. Resource value was manipulated along the dimension of accessibility to the protagonist. In the low-value condition, the protagonist has access to the item or experience frequently or infrequently. In the high-value condition, the protagonist has a one-time-only (and thus extremely infrequent) opportunity to access the resource. There were two versions of the low-value resource script, and an example of each follows:

Cara brought a very special treat with her to the park. This is her favorite treat, and it is very delicious. *It is very hard to find and Cara hardly ever gets to eat this treat.* Who do you think Cara would like to share her treat with?
Cara brought a very special treat with her to the park. This is her favorite treat, and it is very delicious. *It is very easy to find and Cara eats this treat a lot.* Who do you think Cara would like to share her treat with?

An example of the high-value resource script is:

There is going to be a special day at the park with a visiting carnival that has lots of fun treats, games, and rides, just like this special cupcake that Cara has. The carnival will only be there for one day, and Cara can only bring along one person with her, so the person she chooses gets to go and enjoy the treats, games, and rides, but the person she does not choose never gets to go. Who do you think Cara would like to invite along with her?

In the low-value resource conditions, the drawings of the cupcake, seashell, and banana are the objects to be shared in the short vignette, so the participants are encouraged to give the object to the one they believe the protagonist would choose. In the high-value resource condition, the protagonist got to bring along one person to the once-in-a-lifetime experience, and the drawings were described as tickets, and participants were encouraged to give the item to the one they thought the protagonist would like to bring along, thus they need not know the word “ticket” to still understand they give the item to the one she preferred to bring along.

Participants thus used the drawing of the resource to help the protagonist chose a preferred recipient. After their choice, the resource was moved behind the storybook, and a new block began with the protagonist in a new scenario.

### Results

To test children’s understanding of kin, friend, and stranger relations as well as their expectations for interactions among people in these relations, their responses to each question and their choice for resource sharing were analyzed using a binomial distribution. Children’s answers to the comprehension questions were coded as a 0 (incorrect) or 1 (correct). For the resource distribution questions, children scored a 1 for choosing to share with the predicted character: sibling in sibling vs. stranger, friend in friend vs. stranger, and sibling in sibling vs. friend.

#### Conceptual Understanding Questions

For conceptual understanding questions, we first analyzed children’s correct responses within each recipient pair using their average score on the two questions per recipient pair. A 2 (age group) by 3 (recipient pair) repeated measures ANOVA revealed significant main effects of recipient pair, *F*(2,188) = 16.1, *p* < 0.001 and age group, *F*(1,94) = 12.72, *p* = 0.001. There was no significant interaction. Follow-up analyses comparing children’s accuracy by age group revealed that 4-year-old children answered with greater accuracy than 3-year-old children, *t*(94) = 3.55, *p* = 0.001. Additional analyses comparing children’s responses across recipient pairs revealed that children answered more questions correctly for kin vs. stranger and friend vs. stranger than for kin vs. friend [*F*(1,94) = 16.56, *p* < 0.001; *F*(1,94) = 25.92, *p* < 0.001], but they did not show different performance when comparing kin vs. stranger to friend vs. stranger questions, *F*(1,94), = 1.21, *p* = 0.27.

Next, children’s responses on each question were analyzed separately by age using one-sample two-tailed *t*-tests, with chance performance at 0.5 as they chose between two characters with one correct answer (**Table 1**). Correcting for multiple comparisons using Bonferroni correction for two questions in each test block, *p*-values should be considered significant when *p* < 0.025 for these analyses; all *p*-values are given in **Table 1**. Four-year-old children answered correctly on both questions for the sibling vs. stranger, and 3-year-old children answered one correctly (never seen) and one incorrectly (last name). Children at 3- and 4-years-old answered both questions correctly for friend vs. stranger. However, all children erred on questions contrasting sibling with friend.

**Table 1:** Children’s conceptual understanding responses in Experiment 1.

	Question	3-year-olds		4-year-olds	
Kin vs. Stranger	Which has the same last name as X?	ns	*p* = 0.042	^∗∗∗^K	*p <* 0.001
	Which has X never seen before?	^∗∗∗^S	*p <* 0.001	^∗∗∗^S	*p <* 0.001
Friend vs. Stranger	Which has X played with many times?	^∗∗^F	*p* = 0.003	^∗∗∗^F	*p <* 0.001
	Which does X not know much about?	^∗∗^S	*p* = 0.003	^∗∗∗^S	*p <* 0.001
Kin vs. Friend	Which has the same grandparents as X?	ns	*p* = 0.78	ns	*p* = 0.25
	Which could X meet for the first time at school?	ns	*p* = 0.57	ns	*p* = 0.042

*Children provided responses about a sibling (K for kin), friend (F), and stranger (S). Three-year-olds answered one question correctly (^∗∗∗^*P* < 0.001) and one incorrectly for kin vs. stranger, both question correctly for friend vs. stranger (*^∗∗^P* < 0.005), and both questions incorrectly for kin vs. friend. Four-year-olds answered all questions correctly for kin and friend vs. stranger (^∗∗∗^*P* < 0.001) but both incorrectly for kin vs. friend. All *p*-values were corrected for multiple comparisons.*

#### Resource Sharing Questions

Children showed no clear judgments of differential sharing based on resource value: the 2 (resource value) by 3 (recipient pair: sibling vs. stranger, friend vs. stranger, sibling vs. friend) repeated-measures ANOVAs, conducted at each age, revealed no main effects or interactions (all *p*s > 0.05).

**FIGURE 1:**
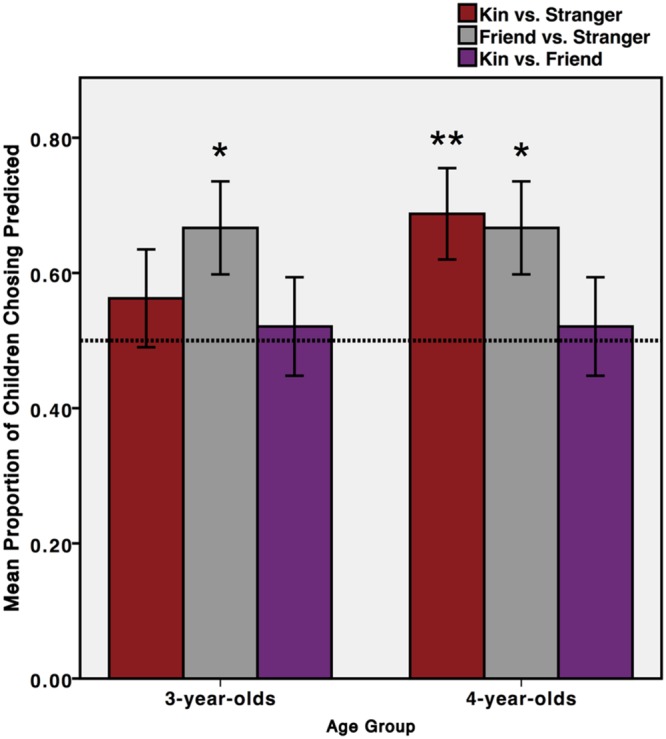
**Children’s sharing choices in Experiment 1.** Children (*n* = 96) allocated a resource to one character in each dyad. Three-year-old children chose to share with a friend over stranger (*^∗^P* < 0.05), and 4-year-old children chose to share with a sibling over stranger (*^∗∗^P* < 0.01) and friend over stranger (*^∗^P* < 0.05). Error bars represent one standard error.

Due to the minimal impact of the resource value manipulation, we collapsed across cost-level and analyzed children’s responses to each test pair using one-sample two-tailed *t*-tests with chance performance set to 0.5 (**Figure 1**). Three-year-olds chose friend over stranger, *t*(47) = 2.42, *p* = 0.019, but not kin over stranger, *t*(47) = 0.86, *p* = 0.39; 4-year-olds chose kin over stranger, *t*(47) = 2.77, *p* = 0.008, and friend over stranger, *t*(47) = 2.42, *p* = 0.019. At neither age did children choose kin over friend or the reverse (both *t*s < | 1|).

#### Additional Analyses

In light of children’s poor performance on the comprehension questions, further analyses focused on the performance of subsets of children who might be expected to have a greater understanding of kinship relations. First we compared the responses in the sharing task for children with the same age or older siblings (*n* = 47) as compared to children with younger or no siblings (*n* = 49). For children at age 3–4 years that have a younger sibling, that sibling is an infant or toddler, so the child-aged sibling in the storybook may not relate as directly to their own experience. Nevertheless, the performance of children in these two categories did not differ, *F*(1,94) = 0.03, *p* = 0.86.

Next, we analyzed the performance of the subset of children who answered all six conceptual understanding questions correctly, *n* = 22 (4: 3-year-olds; 18: 4-year-olds, mean age = 52.63 months). First, we compared these children’s resource sharing choices to children who did not answer all questions correctly, and the 2 (all correct vs. not all correct) by 3 (recipient pair) RM ANOVA revealed a main effect of answering all comprehension questions correctly, *F*(1,94) = 7.05, *p* = 0.009, showing that these children chose in the predicted direction significantly more often. There were no other main effects or interactions. Further analyses showed that children who answered all conceptual understanding questions correctly expected favor to go to kin over stranger, *t*(21) = 3.78, *p* = 0.001, and friend over stranger, *t*(21) = 2.98, *p* = 0.007. They also tended to favor kin over friend, but this tendency was not significant, *t*(21) = 1.79, *p* = 0.088.

### Discussion

Across all levels of resource cost, 3- and 4-year-old children showed explicit understanding and differential expectations for resource sharing between siblings and strangers and between friends and strangers, but not between friends and siblings. Children distinguished well between familiar people, whether friends or siblings, and unfamiliar people. In contrast, children did not show the tested distinctions between familiar people within vs. outside the family. Overall, these findings replicate previous findings that children divide plentiful resources equally between siblings and friends (Olson and Spelke, 2008), in roughly the same manner as they do in the current study with limited resources. We did not find differences across scenarios that varied resource value according to accessibility, though this may indicate either that children are insensitive to cost manipulations or that scarcity, the dimension along which cost was manipulated, does not effectively convey value to children of this age.

We did not find that children with siblings were more likely to favor kin over friends than were other children, though this binary categorization of sibling relations may not be sufficiently sensitive. The quality of a child’s relationship with a sibling might be a better predictor of sharing with kin in the present experiment than the experience of having a sibling. Future research examining individual differences in sibling relationships could explore this possibility further.

Children’s answers to the comprehension questions suggest a different reason for their equal division between siblings and friends: children may be unsure about the conceptual distinction between the two. It is possible, however, that children understood this distinction but had trouble answering the specific questions asked, because they did not understand the term “grandmother” or the significance of surnames. Consistent with this possibility, children who passed all the comprehension questions also failed to show a robust favoring of kin, although they showed a non-significant trend in that direction. Thus, even children who understand the distinction between kinship and friendship may fail to favor kin over friends.

Experiment 2 begins to investigate this possibility in two ways. First, we included more comprehension questions using well-known kin terms such as “mom” as well as questions with no kin terms. Second, we asked children about their own friends and siblings, rather than the friends and sibling of hypothetical characters. Despite children’s failure to favor kin over friends in the present study and in previous studies presenting hypothetical characters (Olson and Spelke, 2008), it is possible that young children would choose to favor kin over non-kin when they consider how they would distribute resources to their own friends and relatives. To test this possibility, Experiment 2 presented children with first-person, hypothetical scenarios involving themselves and their own sibling or friend, as well as a stranger. In each of three scenarios within a story, children were told that they received a resource and were asked how they would distribute it. The cupcake and banana both represented shared activities in the low-resource conditions—eating, and feeding animals together, respectively—but the seashell was given in whole as a gift. In order to better equate the three social scenarios, we replaced the seashell with a shovel in Experiment 2 and modified the social scenario to be about building a sandcastle together, a shared activity.

## Experiment 2

The second experiment investigated children’s expectations for sharing in hypothetical first-person social interactions among their own kin and friends. An experimenter read children an interactive storybook in which they were the protagonist, who traveled to different locations and interacted with their own sibling, their own friend, or a stranger. Children were asked questions about the different relations as well as with whom they would choose to share a resource. We tested children’s conceptual understanding of social relations with additional questions. To specifically test their knowledge of kin compared to non-kin—friends and strangers—the same questions pertaining to kin were asked across pairs with each type of relation. Because 4-year-old children made many errors on the conceptual questions in Experiment 1, we included 5-year-old children in this experiment to compare their performance to that of younger children.

### Materials and Methods

#### Participants

One hundred eight children from the Cambridge and Boston area participated in this study, with 36 children aged 3.5 years (18 female, 36.07–47.57 months, mean age = 42.76 months), 4.5 years (18 female, 48.17–59.97 months, mean age = 53.08 months), and 5.5 years (18 female, 60.17–71.3 months, mean age = 66.07 months). All participants had at least one sibling in order to make the first-person storybook realistic and relevant. Children received a gift for their participation, and parents received a reimbursement for their travel. This study was carried out in accordance with the recommendations of the Committee on the Use of Human Subjects in Research at Harvard University with written informed consent from a parent or legal guardian of all subjects and verbal agreement from participants.

#### Materials

This study used an adapted version of the storybook from Experiment 1 that incorporated the participant as the protagonist. There was one storybook for male and one for female participants. The protagonist in each story was drawn without color in the storybook; participants first colored in a cutout version of the protagonist to represent themselves in the storybook. Participants also selected colored cutout drawings of the additional three characters representing a sibling, friend, and stranger from among six possible characters: three males and three females. Participants with a sibling of their same gender had a storybook with all gender-matched characters. If participants only had one or more siblings of the opposite gender, they could select either gender for a friend, and the stranger was gender-matched to the sibling. Participants chose the two characters that represented a sibling and a friend, and the experimenter added the third character to represent the stranger. These characters were inserted into the story at relevant times using Velcro.

The warm-up scenario, including the introductory and sharing phase involving the cat and dog, was the same as Experiment 1 except that it was narrated such that the participant was the protagonist. The experiment consisted of three test blocks, each with an introductory phase followed by a sharing phase. In all three scenarios, the sharing phase involved both a shared object and a shared activity. The drawings of the resources were the same as in Experiment 1 except that a shovel now replaced the seashell, which did not lend itself readily to a shared activity.

#### Procedure

This study began with participants coloring in a picture of a boy or girl to represent them, which they then used in order to pretend that they were in the storybook. Once children had finished coloring, they were told that other people they knew would also be in the storybook. The experimenter then presented children with drawn, laminated pictures of three boys or girls, depending on the gender of their sibling. The experimenter obtained sibling information from the parents or guardians prior to the study during the consent process. Children were encouraged to choose one picture to be their sibling in the story. Children with multiple siblings were encouraged to choose one to be in the storybook. After children made a selection, they were told that a friend would be in the storybook too, and they were asked to choose from one of the remaining pictures. If children had a gender-matched sibling, they also had a gender-matched friend and stranger. If children had a sibling of the opposite gender, they were allowed to choose a friend of either gender, but the stranger was matched to the gender of the child’s sibling. The three pictures that represented the sibling, friend, and stranger were incorporated into the storybook by sticking them onto the pages using Velcro.

Once children had the three pictures chosen and their picture colored for themselves, the experimenter began the story. The first page showed the same character they had colored in, and they were invited to pretend that they were in the storybook. Children were encouraged to place their drawing into the storybook. The story then progressed through the warm-up sequence and practice trial as in Experiment 1 with adjustments in narration to render the story as a first-person narrative.

The test blocks consisted of the protagonist, in this case the participant, in a new scenario and interacting with two of the three other characters: a sibling, a friend, and a stranger. As in Experiment 1, the introductory phase consisted of showing the protagonist at the new location and discussing that new place. Then, the protagonist was shown with two of the characters, children were reminded of who they were (“your sister/brother,” “your friend,” “a girl/boy you have never met before”), and children were asked questions about these people. Each question was followed by, “Would it be [X] or [Y]?” with the experimenter pointing and labeling the two options by their relationship to the child. Children were asked the same two questions that were used in Experiment 1 during each test block, rephrased into first-person questions, as well as two additional questions. The new questions were: “Which has the same mom as you?” “Which has a different mom than you?” “Which lives in the same house as you?” “Which lives in a different house than you?” The added questions for friend and stranger were: “Which would you invite to your birthday party?” and “Which have you never invited over to play before?” Because the kin concept questions were of specific interest, the questions in the kin and friend as well as the kin and stranger pairs were counterbalanced across children. Question order within test blocks and order of relation pairs were also counterbalanced across participants.

For the sharing phase of each test block, children were shown their own protagonist character with a newly acquired resource: a cupcake, a shovel, or a banana. Children were then told they had one additional item that they could share with one of the two people there with them. The cupcake was described as a treat to eat at the park. Children were told the shovel could be used to build a sand castle with the person they choose, and the banana was for feeding animals at the zoo, and they could bring one person along with them to feed the animals. As in Experiment 1, participants received a drawing of the item and were encouraged to give it to their chosen recipient in the story. After they made a decision, the item was placed behind the storybook, and the story proceeded to the next test block.

### Results

**FIGURE 2:**
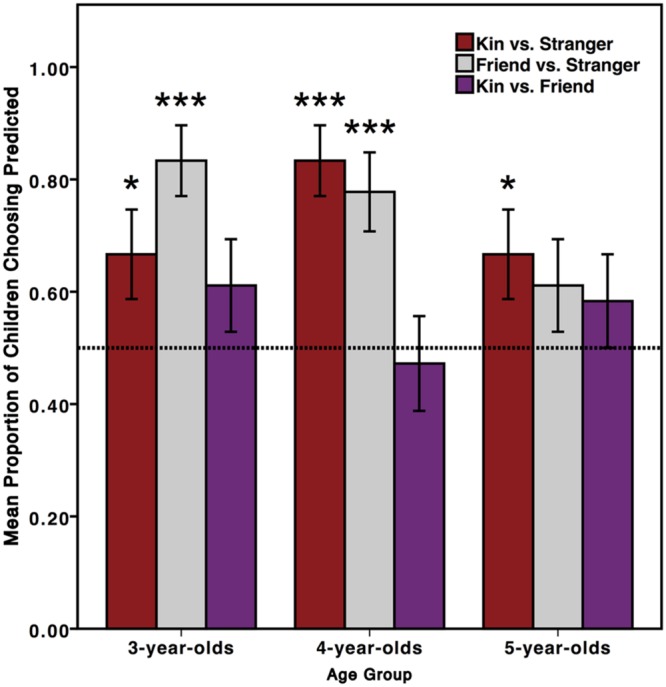
**Children’s sharing choices in Experiment 2.** Children (*n* = 108) allocated a resource to one character in each dyad. Three- and 4-year-old children chose to share with a sibling over stranger and a friend over stranger (*^∗^P* < 0.05; *^∗∗∗^P* < 0.001). Five-year-old children chose to share with a sibling over stranger (*^∗^P* < 0.05). Error bars represent 1 standard error.

The same analyses were conducted for Experiment 2 as for Experiment 1. For the resource distribution, children scored a 1 for choosing to share with the predicted character: sibling rather than stranger, friend rather than stranger, and sibling rather than friend. For comprehension questions, children’s answers were coded as a 0 (incorrect) or 1 (correct). We used two-tailed one-sample *t*-tests to test whether children’s responses were significantly above chance performance of 0.5 (**Figure 2**). This experiment included storybooks with the child as the protagonist and their own siblings and friends (vs. all female characters in Experiment 1). We thus included sex as a variable in the analyses to test for potential sex differences.

#### Conceptual Understanding Questions

For conceptual understanding questions, we first analyzed children’s accuracy for questions within each recipient pair using their average score on the four questions. A 3 (age group) by 3 (recipient pair) repeated measures ANOVA analyzing their responses revealed a significant main effect of age group, *F*(2,105) = 23.32, *p* < 0.001, showing increasing accuracy with age, and no other main effect or interaction. Follow-up analyses comparing children’s accuracy by age group revealed a significant difference in responses between 3- and 4-year-olds, *t*(105) = 3.24, *p* = 0.002, between 3- and 5-year-olds, *t*(105) = 6.82, p < 0.001, and between 4- and 5-year-olds, *t*(105) = 3.61, *p* < 0.001.

Next, children’s responses at each age were analyzed for each question using two-tailed one-sample *t*-tests with chance performance at 0.5 as they chose between two characters with one correct answer (**Table 2**). To correct for multiple comparisons using Bonferroni correction for four questions in each test block, *p*-values should be considered significant when *p* < 0.0125 for these analyses; all *p*-values are given in **Table 2**. All questions asked for kin vs. stranger were also asked for kin vs. friend for different children.

**Table 2:** Children’s conceptual understanding responses in Experiment 2.

	Question	3-year-olds		4-year-olds		5-year-olds	
Kin vs. Stranger (*n* = 18 per question)	Which has the same last name as you?	ns	*p* = 0.057	ns	*p* = 0.16	^∗∗^K	*p* = 0.002
	Which have you never seen before?	^∗∗^S	*p* = 0.002	^∗∗^S	*p* = 0.002	^∗∗∗^S	*p* < 0.001
	Which lives in the same house as you?	^∗∗∗^K	*p* < 0.001	^∗∗∗^K	*p* < 0.001	^∗∗∗^K	*p* < 0.001
	Which lives in a different house than you?	^∗∗^S	*p* = 0.002	ns	*p* = 0.014	^∗∗∗^S	*p* < 0.001
	Which has the same grandparents as you?	ns	*p* = 1.0	^∗∗^K	*p* = 0.002	^∗∗∗^K	*p* < 0.001
	Which could you meet for the first time at school?	ns	*p* = 0.65	^∗∗^S	*p* = 0.002	^∗∗∗^S	*p* < 0.001
	Which has the same mom as you?	ns	*p* = 1.0	ns	*p* = 0.014	^∗∗∗^K	*p* < 0.001
	Which has a different mom than you?	ns	*p* = 0.65	ns	*p* = 0.014	^∗∗∗^S	*p* < 0.001
Friend vs. Stranger (*n* = 36)	Which have you played with many times?	^∗∗∗^F	*p* < 0.001	^∗∗∗^F	*p* < 0.001	^∗∗∗^F	*p* < 0.001
	Which do you not know much about?	ns	*p* = 0.017	^∗∗∗^S	*p* < 0.001	^∗∗∗^S	*p* < 0.001
	Which would you invite to your birthday party?	^∗^F	*p* = 0.006	^∗∗∗^F	*p* < 0.001	^∗^F	*p* = 0.006
	Which have you never invited over to play?	ns	*p* = 0.32	^∗∗∗^S	*p* < 0.001	^∗∗∗^S	*p< 0.001*
Kin vs. Friend (*n* = 18 per question)	Which has the same last name as you?	ns	*p* = 1.0	ns	*p* = 0.057	^∗∗∗^K	*p* < 0.001
	Which have you never seen before?	ns	*p* = 0.16	ns	*p* = 0.16	^∗∗∗^F	*p* < 0.001
	Which lives in the same house as you?	ns	*p* = 0.16	^∗∗^K	*p* = 0.002	^∗∗∗^K	*p* < 0.001
	Which lives in a different house than you?	ns	*p* = 0.057	^∗∗^F	*p* = 0.002	^∗∗∗^F	*p* < 0.001
	Which has the same grandparents as you?	ns	*p* = 0.65	ns	*p* = 0.65	^∗∗∗^K	*p* < 0.001
	Which could you meet for the first time at school?	ns	*p* = 1.0	ns	*p* = 0.16	^∗∗^F	*p* = 0.002
	Which has the same mom as you?	ns	*p* = 0.65	ns	*p* = 0.057	^∗∗∗^K	*p* < 0.001
	Which has a different mom than you?	^∗∗^F	*p* = 0.002	^∗∗∗^F	*p* < 0.001	^∗∗∗^F	*p* < 0.001

*Children provided responses about a sibling (K for kin), friend (F), and stranger (S). Three- and 4-year-olds answered only some questions correctly, and 5-year-olds demonstrate a clear understanding of the distinctions between relations in answering all questions correctly (^∗^*P* < 0.0125; *^∗∗^P* < 0.0025; ^∗∗∗^*P* < 0.001; corrected for multiple comparisons).*

Three-year-olds answered three questions correctly for kin vs. stranger that were answered incorrectly when asked about kin vs. friend, and they correctly knew one answer for kin vs. friend but not kin vs. stranger (**Table 2**). Four-year-olds were correct on half of the questions for kin vs. stranger but only two questions for kin vs. friend. Five-year-olds answered all of the questions correctly.

In questions about a friend vs. stranger, 3-year-olds answered two questions correctly (played with, birthday) and two incorrectly (not know, never invited). Four- and 5-year-olds were correct in answering all questions.

#### Resource Sharing Questions

Three- and 4-year-old children chose to share with siblings over strangers [*t*(35) = 2.092, *p* = 0.044; *t*(35) = 5.29, *p* < 0.001] and with friends over strangers [*t*(35) = 5.29, *p* < .001; *t*(35) = 3.95, *p* < 0.001] but not with siblings over friends (*p*s > 0.05). Five-year-old children chose to share with siblings over strangers, *t*(35) = 2.092, *p* = 0.044, but not with friends over strangers or with siblings over friends (*p*s > 0.05).

The 2 (sex) by 3 (age group) by 3 (recipient pair: sibling vs. stranger, friend vs. stranger, sibling vs. friend) repeated-measures ANOVA on the measure of children’s resource sharing preferences revealed a main effect of recipient pair, *F*(2,204) = 6.82, *p* = 0.001, showing that children chose to share differentially depending on the social contrast. Follow-up analyses comparing children’s choices for each recipient pair revealed their stronger sharing preference for the predicted character (sibling in sibling vs. stranger and sibling vs. friend; friend in friend vs. stranger) in sibling vs. stranger, *F*(1,102) = 8.58, *p* = 0.004, and friend vs. stranger, *F*(1,102) = 9.39, *p* = 0.003, as compared to sibling vs. friend, but their sharing preferences did not differ between sibling vs. stranger and friend vs. stranger, *F*(1,102) = 0.15, *p* = 0.70. There was a significant recipient pair by age by sex interaction, *F*(4,204) = 3.82, *p* = 0.005, showing that boys and girls demonstrated different sharing patterns according to recipient as they grow. Three-year-olds show sex differences in preference for two recipient pairs (sibling vs. stranger and sibling vs. friend), whereas 4-year-olds differ by sex for only one recipient pair (friend vs. stranger), and 5-year-olds do not differ for any recipient pair (Supplementary Table S1 in Supplementary Material).

#### Additional Analyses

To look further into the findings for children who answered all questions correctly in Experiment 1, 3- and 4-year-old children who answered a majority of conceptual understanding questions correctly (10 of 12) in this experiment were analyzed for their sharing choices. Only 9 of those children answered all questions correctly, but there were twice as many questions as Experiment 1, so the criteria for correct responses was relaxed. Children with 10 of 12 correct, *n* = 28 (7: 3-year-olds; 21: 4-year-olds, mean age = 50.8 months) expected favor to go to kin over stranger, *t*(27) = 6.60, *p* < 0.001, and friend over stranger, *t*(27) = 4.36, *p* < 0.001, but not to kin over friend, *t*(27) = 0.37, *p* = 0.71. Thus, Experiment 2 failed to confirm the non-significant trend toward a preference for kin over friend shown by children who passed all the comprehension questions in Experiment 1.

### Discussion

Overall, young children showed a preference to share with their own siblings and friends over children they had never met before, but they shared roughly equally with their own sibling and friend. Three- and 4-year-old children showed a similar pattern of sharing with their own relations in Experiment 2 as they did in third-party scenarios in Experiment 1, except that 3-year-olds now also chose to share with a sibling over a stranger. Although 3- to 4-year-old children continued to make some errors on the comprehension questions, failures of comprehension do not account for their failure to favor kin over strangers.

Five-year-old children showed weaker patterns: They expressed a significant but small preference for sharing with their own siblings over strangers and no preference for sharing with friends over strangers or with siblings over friends. In general, five-year-old children showed less preferential sharing with known over unknown social partners. This finding may result from the new social environments such children encounter as they start school and interact with unfamiliar children whom they are encouraged to treat fairly and nicely—in this experiment, children similar to the stranger. Though the present study did not collect information on children’s school experience, future research could address whether the difference in performance between younger and older children in this study was related to school experience. At 5 years, children’s performance on the comprehension questions revealed the clearest understanding of the distinction among the three types of relationships, even as children’s performance on the resource distribution questions suggested a de-emphasis of these distinctions in sharing contexts.

Experiment 2, like Experiment 1, provided no evidence for an in-group bias toward a family member over a non-family friend, suggesting children do not consider family to be a privileged in-group, even when children make resource-sharing decisions about their own siblings. However, this experiment tested only one domain of social interaction: resource sharing. It is possible that children would be more sensitive to family as a group in other social contexts. For example, adults are more likely to favor their close relatives in specific social contexts involving aid in serious times of need (Burnstein et al., 1994). Experiment 3 addressed this question by examining children’s expectations for social interactions across a more diverse set of social settings involving both helping and sharing.

A further limitation of Experiments 1 and 2 concerns the use of hand-drawn illustrations to represent people. Even though the children in Experiment 2 were asked to pretend that they and their actual friends and siblings were participants in the story, the depicted scenarios may not have been compelling in demonstrating cues to relatedness. In the next experiment, pictures of actual children were used and faces were morphed such that siblings resembled one another. With this manipulation, we return to the third-party narrative structure of Experiment 1 and ask whether children expect other children to favor kin over non-kin in sharing and giving contexts.

Experiment 3 investigated further children’s expectations for social interactions across multiple contexts to see whether their preferences from the first-person scenarios of Experiment 2 replicated or differed when children were presented with a third-person task with enhanced cues to kinship and a wider range of social scenarios. We focused primarily on the relationship comparison for which children did not show a clear preference to favor one person over the other: sibling vs. friend.

## Experiment 3

In this Experiment, we tested 3- 4-, and 5-year-old children’s expectations for third-party social interactions between siblings and friends, as in Experiments 1 and 2, using new cues to kinship and new methods. We used face morphology software to present more compelling cues to sibling relations in a third-party context. Given the stable developmental improvement in conceptual understanding questions presented in previous experiments, we did not include any conceptual understanding questions and instead added additional questions regarding expectations for social interactions. Additional social scenarios were added because children did not show strong preferences in resource sharing contexts in the previous experiment, and adults have demonstrated kin preference in specific contexts that call for more costly help (Burnstein et al., 1994; Essock-Vitale and McGuire, 1985). Rather than manipulate resource cost, we presented contexts that called for helping or sharing, asking whether children thought the protagonist character would more readily come to the aid of a sibling than a friend.

### Materials and Methods

#### Participants

Forty-eight Cambridge and Boston area children participated in this study: 16 3.5-year-olds (8 female, mean age = 41.87 months), 16 4.5-year-olds (8 female, mean age = 52.73 months), and 16 5.5-year-olds (8 female, mean age = 66.45 months). All participants had at least one sibling. Children received a gift for their participation, and parents were given a travel reimbursement. This study was carried out in accordance with the recommendations of the Committee on the Use of Human Subjects in Research at Harvard University with written informed consent from a parent or legal guardian of all subjects and verbal agreement from participants.

#### Materials

We presented children with two different sets of faces on a computer, described as a protagonist, his or her brother or sister (henceforth, sibling), and his or her friend. The stimuli consisted of photographs of real children, one of which was created using morphing, so to maintain high quality of the images, stimuli was presented on a computer, though the vignettes were still presented to children like stories. For each trial, children were introduced to three characters and explicitly told how the central character was related to the other two characters (as a friend or sibling). Then children were asked whom they thought the central character might prefer to interact with across four different prosocial scenarios involving helping and sharing in the context of short vignettes.

#### Procedure

Children were told they were going to hear some stories. For each trial, three children’s faces appeared on the screen with the protagonist in the center and two individuals on either side of the protagonist. One character’s image had been created by morphing the protagonist’s face with a third, unseen child’s face such that the character resembled the protagonist as a sibling would. Within a trial, all three characters were of the same sex. There were two sets of characters, one set of girls and one of boys, each presented in two trials for a total of four trials. Two trials involved prosocial interactions resembling sharing—the protagonist could give one recipient a cookie or lend one recipient a bike. The other two trials involved helping: the protagonist could assist one recipient in completing a puzzle or math homework (See Supplementary Materials for full vignettes). Each triad of characters—the protagonist and the two relations—was presented on two trials. The orders of picture sets and test questions were counterbalanced across children as well as which side the sibling was on and which character’s image had been morphed to be the sibling. Two more sets of characters appeared on four additional trials testing other social comparisons, but we do not present their findings here, because they always followed the present trials and their findings are not readily interpretable (see the Supplementary Materials).

The experimenter introduced the protagonist first by name, pointing at the central picture, and then introduced the first outer character by name, pointing to his or her picture. Then, children were told the two characters had a lot in common and were given one other piece of information about their relationship (e.g., they went to the same school (friend) or lived in the same house (sibling)). Next, children were told how the characters were related: respectively, as friends or as brothers/sisters. Finally, children were introduced to a hypothetical scenario in which the protagonist had to make a decision as to whom he or she would choose to share with or help. An example was:

One day at school, Timmy is working on a dinosaur puzzle and Charlie is working on a train puzzle. Peter likes dinosaurs and trains. Who do you think Peter will help with their puzzle – Timmy or Charlie?

Children were encouraged to point to the picture of the child they thought the protagonist would choose. After making a choice, the experimenter proceeded to the next trial and introduced or reintroduced the next set of characters. When characters were reintroduced a second time in new stories, the experimenter would remind children of who each character was and how the protagonist was related to the other two characters while pointing to each one. For example:

Do you remember Peter? Peter and Charlie both live in the same house. They are brothers. Peter and Timmy both go to the same school. They are friends.

Next, a new hypothetical scenario was introduced and children were asked how they thought the protagonist would behave. The vignettes were presented in one of four counterbalanced orders.

### Results

Children’s responses on each trial were coded as choosing the sibling (1) or friend (0). The 3 (age group) by 4 (question) repeated-measures ANOVA on children’s selections revealed no significant main effects or interactions (all *p*s > 0.05), though there was a marginal main effect of age group, *F*(2,45) = 2.42, *p* = 0.10.

**FIGURE 3:**
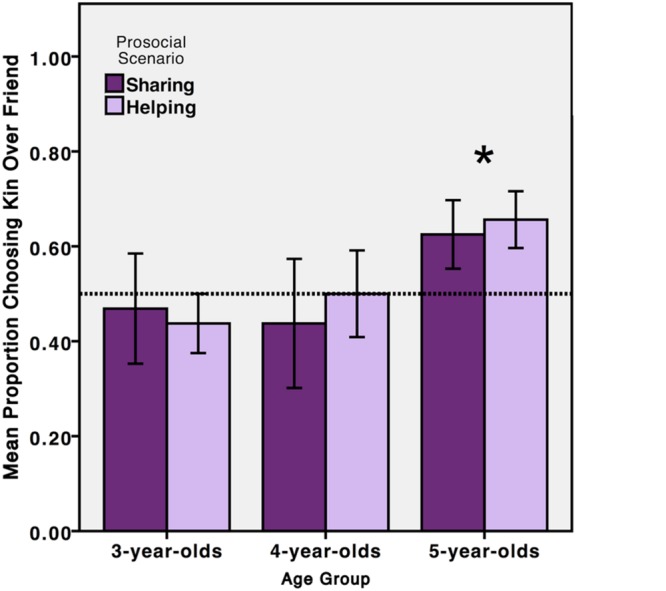
**Children’s social choices in Experiment 3.** Three- and 4-year-old children (*n* = 32) did not show a clear social preference between sibling and friend, but 5-year-old children (*n* = 16) did choose for the protagonist in the vignettes to favor a sibling over friend (*^∗^P* < 0.05). Children’s responses followed the same pattern for both sharing and helping prosocial scenarios. Error bars represent one standard error.

Each age range was then analyzed for an overall preference for sibling over friend. Children’s four choices were summed and analyzed using a two-tailed, one-sample *t*-test with chance performance set to 0.5 (**Figure 3**). Children did not expect protagonists to choose their siblings over their friends at three years of age (*M* = 0.45, *SD* = 0.26), *t*(15) = –0.72, *p* = 0.49, or four years of age (*M* = 0.44, *SD* = 0.31), *t*(15) = –0.81, *p* = 0.43. Five-year-olds did expect the protagonists to favor kin over friends (*M* = 0.63, *SD* = 0.22), *t*(15) = 2.24, *p* = 0.041.

### Discussion

Despite increasing the salience of sibling relations using facial morphology, 3- and 4-year-old children did not expect preferential prosocial behavior toward siblings over friends. In contrast to younger children and to the findings of Experiment 2, however, 5-year-old children now expected the protagonist to favor their sibling, with a non-significant trend suggesting that an expectation of prosocial behavior toward kin seems to develop by around 5 years. The presence of shared face morphology between the sibling characters may have increased the salience of the sibling relation at this age. Alternatively or in addition, the inclusion of more social contexts may have triggered an increased expectation for favoring kin in 5-year-old children. Younger children may not yet have a clear understanding of kinship or friendship, or, alternatively, they may understand distinctions among these relations but not have robust sharing preferences among them.

## General Discussion

We found that 3-, 4-, and 5-year-old children overall showed a preference for sharing with siblings and friends over strangers (children they have never met), but they did not have a strong preference between a sibling and a friend. Children expected the same sharing behaviors when distributing limited resources as they do for plentiful resources, replicating past research (Olson and Spelke, 2008). Thus, children did not privilege family members as an in-group, or at least not in resource sharing scenarios comparing family members (siblings) to known social partners outside the family (friends) (Experiments 1 and 2). When siblings are made more salient and social contexts were more varied, 5-year-olds, but not 3- and 4-year-olds, did expect third party protagonists to favor their siblings over their friends (Experiment 3).

Children’s understanding of these social pairs: sibling and friend, sibling and stranger, friend and stranger, improved with age, as 5-year-old children’s performance revealed a clearer understanding of the distinction among the three types of relationships than 3- and 4-year-olds’. Children showed good understanding for friend vs. stranger contrasts by ages 3 or 4 (Experiment 1 and 2, respectively) and good understanding for sibling vs. stranger contrasts by ages 4 or 5 (Experiment 1 and 2, respectively). For sibling vs. friend contrasts, only 5-year-old children answered most questions correctly.

Five-year-old children’s performance revealed a clearer understanding of the distinction among siblings, friends, and strangers, even as their resource distribution beliefs suggest a de-emphasis of these distinctions in sharing contexts. At least when sharing scenarios are depicted in illustrated storybooks, specifically, the oldest children were less biased against sharing with strangers.

Although children organize their social world in a variety of ways, grouping individuals by gender, accent or language, and race, the present studies suggest that family relations are not clear to children from a young age. Children distinguish people who are socially related to one another (family members, friends, neighbors) from those who are not (strangers) before they understand the types of relations that connect the people they know. These distinctions begin to emerge at five years in the present studies.

By 5 years, but not before, children start to expect in-group benefits to be given to kin over familiar and valued non-kin, friends. Several factors could explain the late emergence of this expectation. Children may only develop an explicit kinship bias once they have a clear understanding of what constitutes kin vs. non-kin. The comprehension questions given in Experiment 2 provide evidence that children do not clearly differentiate between friends and siblings conceptually until around age 5. This differentiation may be necessary in order to expect favor to go to kin. Prior to age 5, children confuse familiar relations like friends and family, and thus they may expect favor to go to either party.

Young children differentiate how they share based on the recipient, but they do not show much evidence for a kinship bias until around age five, and even then, it is not a robust preference. In both third- and first-person hypothetical sharing contexts, children expected favor to go to familiar others like friends and siblings over strangers, even with a limited resource. However, at 3 and 4 years, children did not demonstrate a clear expectation for whether a sibling or friend should be privileged when choosing between the two. Young children expected equal treatment of siblings and friends in the distribution of resources that are plentiful (Olson and Spelke, 2008) or scarce (Experiments 1–3). In the present studies, the failure of young children to differentiate between friends and siblings is observed not only when resources have minimal value but when their value is increased, and not only when the story presents characters that are unknown to the child, but also when it depicts the child and his or her own sibling and friend in a hypothetical social scenario (Experiment 2).

Adults are more likely to rely on close kin when needing more costly help (Essock-Vitale and McGuire, 1985; Burnstein et al., 1994), but children are not sensitive to the manipulation of cost presented here. This negative finding may indicate either that children are insensitive to cost manipulations or that the experiment failed to manipulate cost effectively for children.

Thus, the present studies demonstrate that an ability to distinguish kin from non-kin emerges slowly over development in these verbally presented, explicit social scenarios, with no evidence for an early emerging ability even in contexts where the stakes are higher or more relevant to the children. However, these findings do not rule out that children may be sensitive to kin distinctions in different circumstances or with different presentations of kin and social relations.

Children’s resource sharing with siblings may not be the best measure of kinship preference, because their allocation of resources to siblings may depend on additional factors such as the quality of their relationship or age difference. Sibling competition over resources may also lead them to prefer to share with a friend over a sibling, as according to theories on sibling rivalry, full siblings compete so long as the benefits outweigh the costs two to one (Hamilton, 1964). Future research could examine whether children show preference for siblings in other areas or whether they show preference for other types of family relations, such as parents. However, this study would need to find a relevant match in familiarity and age (as friend was to sibling) for child-adult relations that are not parents (e.g., nanny or teacher). The present studies do not rule out a preference for kin but do demonstrate that children do not have a robust preference for all kin over non-kin, as they do not robustly share with siblings over friends.

In addition to their ambiguity about sharing, 3- and 4-year-old children show some confusion about what defines sibling vs. friend relationships. By the time children are 5 years old, they demonstrate a better understanding of each relationships, and they also show biases for family over others in some social contexts (Experiment 3), although not in all contexts (Experiment 2).

Why do children confuse friends and siblings when answering questions like, “Who has the same mom as you?” One answer may be that children do not have a clear representation for each type of relationship: friend, sibling, stranger, and that this develops with more experience as they grow. Alternatively, children may have clear representations but lack the vocabulary to demonstrate their understanding. For example, children need to understand words like “same” and “different”, and kinship terms like “grandparent” to answer questions correctly. Though Experiment 2 presented additional comprehension questions to the battery from Experiment 1 to better assess children’s understanding, further research into how well they understood the kin terms and the questions we presented could help distinguish between these explanations.

Another possibility is that children may have representations for these relations, but the specific cues they use to identify and distinguish between them do not work as effectively in modern society. If children distinguish family from friends on the basis of the information that was most reliable in our evolutionary past, one should consider how human groups and social relations were organized then in order to know how to define the groups. One theory posits that family members were defined by communal sharing relationships, in which members are treated as equally and share benefits altruistically among members (Fiske, 1992). However, friends in modern times also show many features that once defined only family relations. For instance, a child is likely to have their friends over to their house, share food with them, and see their own parents acting in a nurturing and protective manner toward their friends. These are all behaviors that children would have only seen directed toward siblings and other family members historically. Thus, what defines the idea of a sibling for a small child may be activated by the type of relationship they have with their friends now. The line between friend and sibling may be blurred in their experience and conceptions, and the distinction may not become explicit and clear until around 5 years of age, when further experience, likely including school experience, may start to clarify these social group boundaries. Before then, both may be seen as communal sharing relationships.

Children also have vastly greater social experiences to drawn upon at age five relative to age three. Socialization pressures could influence 5-year-old children’s sharing preferences in encouraging them to reach out to increasingly to new children around their age, in these experiments, strangers. Children are encouraged to share with others not only in their homes but also in contexts with children they may not have met before including schools, museums, daycare, parks, playgrounds, and other public places where they interact with new children. The present experiments were conducted in a lab environment, which may share many features with these other environments—for example, the experimenter is an unfamiliar friendly adult much like a teacher or children’s museum guide—and thus, the lab may elicit socialized patterns of sharing.

A broader aversion to strangers in early childhood could also be driving young children’s more robust preferences for familiar others to strangers compared to that of 5-year-olds. Most parents encourage their children to avoid strangers, and children of all ages may adhere to this advice equally, however, older children may have different definitions of what individuals may count as a caution-warranting stranger. More specifically, a child their own age that they may not have met before would not be the type of stranger their parents warn them about. Moreover, they may recognize certain contexts in which they should be more or less wary—schools and playgrounds may be safer than airports, parking lots, or amusement parks. Younger children may not have the social experience or skill to differentiate between varying people and contexts, and thus they show a stronger aversion to strangers in our storybook contexts.

Adults’ tendency for altruism toward kin is mediated by emotional closeness (Korchmaros and Kenny, 2001). This finding raises the possibility that children’s equal sharing with friends and family is influenced by this variable as well. Though emotional closeness may have been a factor when children were considering their own relations (Experiment 2), it is less likely to be a factor in the hypothetical third-person scenarios used in Experiments 1 and 3. It is possible, however, that children considered their own relations when making decisions in third party scenarios, and thus emotional closeness or quality of relationship with siblings or friends may still have played a role. Future research could measure or manipulate emotional closeness or relatedness as potential mediating factors in children’s sharing behavior in order to further investigate whether the present findings could be influenced by such factors.

Even if children made no clear distinction between kin and friends in the present experiments, it is possible that they distinguish kin from friends in other contexts. Future research using more sensitive measures of a potential in-group kinship preference, such as implicit measures, might reveal such distinctions. In the present experiments, children are asked explicitly whom they think a character will share with or whom they would like to share with. Adults show a tendency to favor kin in an explicit context as well as a more implicit measure, such as the amount of time they are willing to hold a physically challenging position in order to win money for someone (Madsen et al., 2007). In that case, adults unknowingly hold the position longer for those more closely related to them. A similar study could be conducted with children to see if they put in more effort for kin when they are not as aware of the costs.

These findings raise additional questions for future research. First, is the slow development of understanding of kinship relations a universal feature of human development, or is it specific to children from western, industrialized societies? It is possible that children in traditional societies, in which people live in extended families and emphasize kin relations, come to understand kin relations more precociously. Second, do young children fail to understand any kin relations, or only sibling relations? Children may be able to distinguish their parents from unrelated but highly familiar adults who care for them. However these questions are answered, the present findings shed light on how children are navigating their social worlds and suggest that there is not a sharp, robust in-group boundary that divides kin from non-kin.

## Author Contributions

AS and ES designed the research; AS collected and analyzed the data; and AS and ES wrote the paper. Both authors approved the final version of the manuscript for submission.

## Conflict of Interest Statement

The authors declare that the research was conducted in the absence of any commercial or financial relationships that could be construed as a potential conflict of interest.
