# Supplementary material for: Children’s Expectations and Understanding of Kinship as a Social Category
Source: Front Psychol. 2016 Mar 29;7:440. doi: 10.3389/fpsyg.2016.00440 (PMC4810026; doi:10.3389/fpsyg.2016.00440)
Supplement: Supplementary file 1 [file Table_1.DOCX]

Supplementary Material

Children’s Expectations and Understanding of Kinship as a Social Category

Annie C. Spokes*, Elizabeth S. Spelke

*** Correspondence:** Annie C. Spokes: aspokes@fas.harvard.edu

# Effects of sex on children's judgments

As Supplementary Table 1 reveals, the primary effects found in Experiment 2 are evident in children of each sex, with one exception: 3-year-old boys showed a strong preference for friend over stranger and for kin over friend but no preference for kin over stranger. Both because this pattern was not obtained in Experiments 1 or 3, and because it violates transitivity, the three-way interaction effect likely is spurious.

## Supplementary Table: Further analysis of the three-way interaction between age, sex, and recipient pair for Experiment 2

**Supplementary Table 1. Children’s sharing choices by age and sex.** Children’s sharing choices in Experiment 2 (n = 108), where children allocated a resource to one character in each dyad, separated by age and sex. To look deeper into the significant recipient pair by age by sex interaction, F(4, 204) = 3.82, p = .005, η^2^ = .07, we further analyzed children’s choices in each age group for each sex using two-tailed one-sample t-tests to test whether children’s responses were significantly above chance performance of .5. Children’s answers were again coded as a 0 or 1, with a 1 for choosing to share with the predicted character: sibling rather than stranger, friend rather than stranger, and sibling rather than friend. For each cell, n = 18. (**P* < 0.05; ***P* < 0.01; ****P* < 0.001)

|  | 3-year-olds | | 4-year-olds | | 5-year-olds | |
| --- | --- | --- | --- | --- | --- | --- |
|  | Female | Male | Female | Male | Female | Male |
| Kin vs. Stranger | Kin*  M = .78  p = .014 | ns  M = .56  p = .65 | Kin**  M = .83  p = .002 | Kin**  M = .83  p = .002 | ns  M = .61  p = .36 | ns  M = .72  p = .057 |
| Friend vs. Stranger | Friend**  M = .83  p = .002 | Friend**  M = .83  p = .002 | ns  M = .67  p = .16 | Friend***  M = .89  p < .001 | ns  M = .72  p = .057 | ns  M = .50  p = 1.0 |
| Kin vs. Friend | ns  M = .44  p = .65 | Kin*  M = .78  p = .014 | ns  M = .56  p = .65 | ns  M = .39  p = .36 | ns  M = .61  p = .36 | ns  M = .56  p = .65 |

# Experiment 3 Sample Kin vs. Friend Vignettes

## Sample Kin vs. Friend Vignette 1 (Helping)

This is Peter. Here is another boy that Peter knows. His name is Timmy. Peter and Timmy have a lot in common. They both go to the same school. Peter and Timmy are friends.

Here is another boy that Peter knows. His name is Charlie. Peter and Charlie have a lot in common. Peter and Charlie both live in the same house. Peter and Charlie are brothers.

One day at school, Timmy is working on a dinosaur puzzle and Charlie is working on a train puzzle. Peter likes dinosaurs and trains. Who do you think Peter will help with their puzzle – Timmy or Charlie?

## Sample Kin vs. Friend Vignette 2 (Helping)

Do you remember Peter? Peter and Charlie both live in the same house. They are brothers. Peter and Timmy both go to the same school. They are friends.

Charlie and Timmy sometimes have trouble with their math homework. Peter is really good at math. Peter can stay inside and help one of them with their math homework, but then he will miss recess. Who do you think Peter will help with his math homework – Charlie or Timmy?

## Sample Kin vs. Friend Vignette 3 (Sharing)

This is Sophie. Here is another girl that Sophie knows. Her name is Megan. Sophie and Megan have a lot in common. They both have the same mom. They both have the same dad. Sophie and Megan are sisters.

Here is another girl that Sophie knows. Her name is Erin. Sophie and Erin have a lot in common. They both have the same teacher. They have the same soccer coach. Sophie and Erin are friends.

There was one morning when Megan and Erin both want to borrow Sophie’s bicycle to ride to school. If Sophie lets them borrow it, she will have to walk to school, and it will take a long time. Who do you think Sophie would let ride her bicycle – Megan or Erin?

## Sample Kin vs. Friend Vignette 4 (Sharing)

Do you remember Sophie? Sophie and Erin have the same teacher and the same soccer coach. They are friends. Sophie and Megan have the same mom and dad. They are sisters.

At lunch one day, Sophie has extra cookies in her lunch that she is not going to eat. Who do you think Sophie would give a cookie to – Erin or Megan?

# Additional Questions from Experiment 3: Kin vs. Stranger/Neighbor

Experiment 3 looked further into 3-, 4-, and 5-year-old children's expectations for social interactions across multiple contexts to see whether their preferences from the first-person scenarios of Experiment 2 would replicate or differ when they are given enhanced cues to kinship and are presented with a wider range of social scenarios.

In addition to testing children on kin versus friend scenarios, they were all tested on four additional scenarios comparing two other potential social partners. Five-year-olds were tested on kin versus stranger, due to their less robust social preferences when comparing relations to strangers in Experiment 2. Three- and 4-year-old children were tested on kin versus neighbor. Children at this age consistently expect and chose to share with friends or siblings rather than strangers but do not favor siblings over friends (Experiments 1 and 2; Olson & Spelke, 2008). Previous studies have not addressed whether these distinctions reflect some positive understanding of kinship and friendship (namely, that each of these relationships makes the participants likely to engage in prosocial behavior toward one another) or only a negative understanding of strangers (for example, that strangers are potentially dangerous and should not be followed or spoken to under some circumstances). With an aim of examining kinship rather than stranger avoidance, instead of contrasting siblings to strangers, we contrast siblings to neighbors: children who may be familiar, or if still unfamiliar, associated with broad social groups due to their proximity, but who are not described either as family or as friends.

## Materials and Methods

### Participants

Participants were the same 48 children who participated in Experiment 3.

### Materials & Procedure

In addition to the trials described in Experiment 3, children also answered four questions about another dyad: sibling versus stranger (5-year-olds) or sibling versus neighbor (3- and 4-year-olds). The stranger was described as a child they had never met before. The neighbor was described as a child who lived nearby. Children were asked questions about four social scenarios and asked with whom they thought the central character might choose to interact with in contexts relating to cooperation and protection. The materials and procedure were otherwise the same as Experiment 3, and the four questions were presented in two blocks of two, with each block coming after questions from Experiment 3. The four sibling versus neighbor/stranger vignettes were also presented in one of four counterbalanced orders. Sample vignettes are included below (Section 4).

## Results

Children’s choices on each trial were coded, with a choice of sibling being coded as 1 and stranger/neighbor as 0. The 3 (age group) by 4 (question) repeated-measures ANOVA on children’s selections did not reveal any main effects or interactions (all ps > .05).

Each age range was then analyzed for an overall preference for sibling over either neighbor or stranger. Children’s four choices (1 for sibling, 0 for neighbor/stranger) were averaged and then analyzed using a two-tailed, one-sample t-test with chance performance set to .5. Three-year-olds did not expect preference to go to kin over neighbor (*M =* .47, *SD =* .27), t(15) = -.46, p = .65. Four-year-olds also did not expect preference to go to kin over neighbor (*M =* .5, *SD =* .27), t(15) = 0, p = 1. Five-year-olds did expect preference to go to kin over stranger (*M =* .66, *SD =* .27), t(15) = 2.30, p = .036.

## Discussion

As expected, given the findings of Experiment 2, 5-year-old children expected other children to prefer a sibling to a stranger. Despite increasing the salience of sibling relations using facial morphology, 3- and 4-year-old children did not expect preference toward siblings over neighbors. Children did not give different responses across social scenarios.

Given that children at this age expect preference to a sibling over a stranger, this study suggests that children's differentiation between siblings or friends and strangers in Experiments 1 and 2 may have more to do with their understanding of strangers than with their understanding of kinship or friendship. Alternatively, they may understand distinctions among these relations but not have robust sharing preferences among them.

# Sample Kin vs. Neighbor/Stranger Vignettes

## Sample Kin vs. Neighbor/Stranger Vignette 1 (Protection)

This is Jessica. Here is another girl that Jessica knows. Her name is Molly. Jessica and Molly have a lot in common. A lot of people say that they have the exact same hairstyle. Jessica and Molly are neighbors.

Here is another girl Jessica knows. Her name is Katie. Jessica and Katie have a lot in common. A lot of people say that they have the exact same laugh. Jessica and Katie are sisters.

At recess one day, some older kids started bullying Molly and Katie at different parts of the playground. Jessica wants to go protect one of them from the bullies. Who do you think Jessica would go and protect – Molly or Katie?

## Sample Kin vs. Neighbor/Stranger Vignette 2 (Protection)

Do you remember Jessica? A lot of people say that Jessica and Katie have the exact same laugh. They are sisters. A lot of people say that Jessica and Molly have the exact same hairstyle. They are neighbors.

One day after school, Jessica is waiting for the bus, and it is really cold outside. Katie and Molly are waiting too, but they forgot to wear jackets that day. Who do you think Jessica would share her coat with – Katie or Molly?

## Sample Kin vs. Neighbor/Stranger Vignette 3 (Cooperation)

This is Ben. Here is another boy that Ben knows. His name is Andrew. Ben and Andrew have a lot in common. They both have the same pet dog. Ben and Andrew are brothers.

Here is another boy that Ben knows. His name is David. Ben and David have a lot in common. They both have the same toy cars. Ben and David are neighbors.

At recess one day, Ben wants to play on the swing-set. Ben needs a partner to push him while he swings, and he will push them on their turn on the swings too. Who do you think Ben would like to play on the swings with – Andrew or David?

## Sample Kin vs. Neighbor/Stranger Vignette 4 (Cooperation)

Do you remember Ben? Ben and David both have the same toy cars. They are neighbors. Ben and Andrew both have the same pet dog. They are brothers.

Every year, Ben’s school has a field day with lots of fun games, and Ben needs a partner for this year’s field day. The team that wins the most games wins a big prize and gets to go to an amusement park. Ben really wants to win the prize this year. Who do you think Ben would choose for his partner – David or Andrew**?**
